# Epidemiological and clinical characteristics of malignant melanoma in Southeast Anatolia in Turkey

**DOI:** 10.11604/pamj.2016.24.22.9254

**Published:** 2016-05-06

**Authors:** Bilal Sula, Feyzullah Uçmak, Mehmet Ali Kaplan, Zuhat Urakçi, Mustafa Arica, Abdurrahman Isikdogan

**Affiliations:** 1 Department of Dermatology, Faculty of Medicine, Dicle University, Diyarbakir, Turkey; 2 Department of Gastroenterology, Faculty of Medicine, Dicle University, Diyarbakir, Turkey; 3 Department of Medical Oncology, Faculty of Medicine, Dicle University, Diyarbakir, Turkey

**Keywords:** Malignant melanoma, clinic, epidemiology

## Abstract

**Introduction:**

The present study aimed to establish the epidemiological and clinical characteristics of patients who were histopathologically diagnosed with malignant melanoma (MM).

**Methods:**

The present study retrospectively analyzed the data of 78 patients who were histopathologically diagnosed with MM in Dicle University Medical Faculty, Dermatology and Medical Oncology departments between 2005 and 2014.

**Results:**

The study included 78 patients in total with 44 (56.4%) male and 34 (43.6%) female. Median age of the patients was 62.50 years (range: 27 - 84 years). Of the patients, 78.2% (n = 61) had cutaneous melanoma, 8.9% had solid organ melanoma, and 2.5% had ocular and mucosal melanoma. The most common tumor localization among the patients was the lower extremities with 29.4% (n = 23). The most common histopathological type was nodular malignant melanoma with 35.8% (n = 28). Based on TNM, Clark and Breslow classifications, 26.9% (n = 21) of the patients were stage 4, 26.9% (n = 21) were Clark stage 4, and 37.1% (n = 29) were Breslow stage 4. Median overall survival in all patients was 14.9 months (95% CI 10.9 - 18.8 months). In the multivariate Cox analysis, only stage statistically significantly affecting survival [odds ratio (OR): 0.54; (95% CI 0.16-1.82, p = 0.02)].

**Conclusion:**

Malignant melanoma data are also important for the optimal utilization of effective methods and healthcare resources to prevent the disease. In order to minimize MM mortality and morbidity, not only the society but also physicians from primary and secondary care hospitals should become familiar with melanoma.

## Introduction

Malignant melanoma (MM) is a malignant tumor resulting from melanocytes and nevus cells that are considered to be formed through melanocyte differentiation. MM is the fifth most common cancer in males and the sixth most common cancer in females accounts for 5% of all newly-diagnosed cancers [1]. The estimated lifetime risk for developing invasive melanoma is 1:1,500 in the US. The incidence increases with age, particularly in males. In the US, the melanoma incidence under the age of 40 years is higher in females, whereas it is higher in males above the age of 40 years [1]. The melanoma incidence has shown a significant increase for the last few decades around the world. It exhibits significant differences in race and demographics, and it is one of the most common malignancies in Europe, North America, Australia, and New Zealand, especially in light-skinned individuals. It is more rarely seen in Africans, Latin Americans, Asians and dark-skinned individuals [2, 3]. Australia Queensland is considered the place with the highest MM incidence (51/100,000) in the world. For melanoma incidence, the worldwide annual mean is 3-7% in Caucasians and one- or two-fold increase is estimated once in 10 to 20 years [4, 5]. Seventy-five percent of deaths from skin cancers are due to melanoma. Due to the poor prognosis and fast course, it is also an important public health issue. Despite this, there are few studies demonstrating the epidemiology of cutaneous malignant melanoma in Turkey. A study that was conducted in 2006 in Turkey by collecting data from eight residential areas (Izmir, Eskisehir, Erzurum, Bursa, Edirne, Antalya, Trabzon, and Samsun), reported the melanoma incidence as 1.4/100,000. Based on the data from the Ministry of Health, melanoma incidence was 2.1/100,000 in males and 1.6/100,000 in females in 2009 in Turkey. Additionally, when 2009 data were compared to 2004 data (1.5/100,000 in males and 1.2/100,000 in females), it was seen that MM incidence has increased over the last six years [6, 7]. The present study aimed to establish the epidemiological and clinical characteristics of patients who were histopathologically diagnosed with MM in the Dermatology and Medical Oncology departments of our university and to contribute to MM epidemiology through the data obtained.

## Methods

The present study retrospectively analyzed the data of 78 patients who were histopathologically diagnosed with MM at Dicle University Medical Faculty, Dermatology and Medical Oncology departments between 2005 and 2014. Patient files and pathology reports were analyzed to record age at diagnosis, gender, date of diagnosis, date of exitus, tumor localization, overall survival times, histopathology [based on the American Joint Committee on Cancer (AJCC) classification], Breslow′s tumor thickness, Clark′s level of invasion, spontaneous regression and presence of ulceration, mitotic indices, presence of satellite nodules, capsule invasion and TNM stage. Metastasis regions at diagnosis and follow-up were recorded. Histopathologically, MM was classified as superficial spreading MM, nodular MM, lentigo MM, acral lentiginous MM, and those patients with only a malignant melanoma result on the pathology report were classified as unknown. Overall survival time of the patients was calculated as the time from the diagnosis date to the last control date. Descriptive statistics were expressed in mean ± standard deviation or median (minimum - maximum) for continuous variables, and in case number and (%) for categorical variables. In a comparison of the variables between different groups, the student's t-test was used for continuous variables and Pearson's chi-square test was used for categorical variables. The overall survival was compared by the Kaplan-Meier method and log-rank test. Furthermore, the Kaplan-Meier method was employed again to estimate life expectancy and a 95% confidence intervals relating to each risk factor. All p values were calculated in the two-way analysis and p values < 0.05 were considered statistically significant. All statistical data were calculated in computer environment by a commercial software package SPSS 15.0 (SPSS Inc. Chicago, IL, USA).

## Results

**Figure 1:**
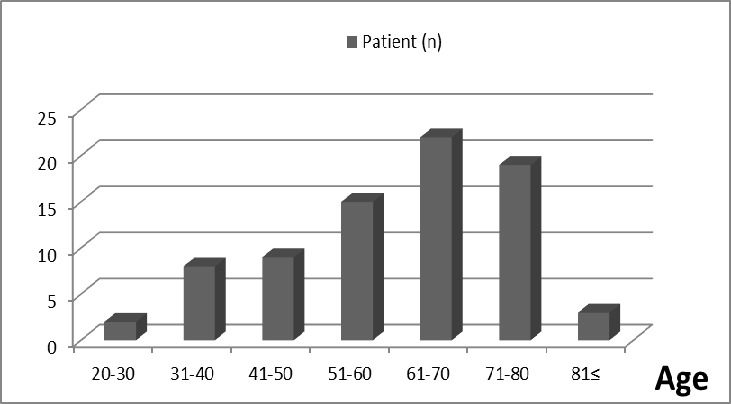
Distribution of patients according to age

**Figure 2:**
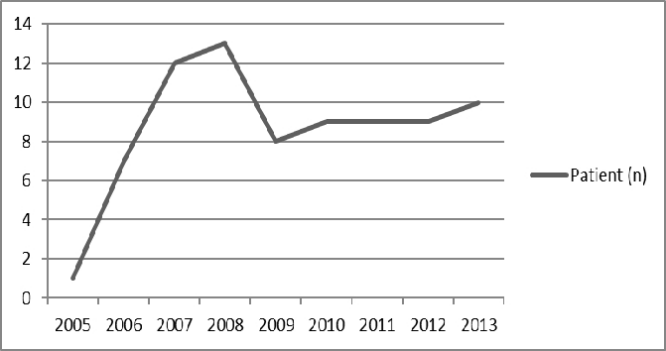
Distribution of patients according to the years

The study included 78 patients in total with 44 (56.4%) males and 34 (43.6%) females. The median age of the patients was 62.50 years (range: 27 - 84 years). The male/female ratio was 1:1.29. When patients were evaluated based on age groups, the most common age range was 61-70 years with 28.2%, as seen in Figure 1. This was followed by the range of 71-80 years (24.4%) and 51-60 years (19.2%). Accordingly, 71.8% of the patients were within 51-80 years. The distribution of MM-diagnosed patients by year between 2005 and 2014 is presented in Figure 2. Of the patients, 78.2% (n = 61) had cutaneous melanoma, 8.9% had solid organ melanoma, 2.5% had ocular and mucosal melanoma, and there were no records accessible for six (7.6%) patients. The most common tumor localization among the patients was lower extremities with 29.4% (n = 23). Based on the frequency, other localizations were the head and neck with 25.6% (n = 20), the trunk with 12.8%, and the upper extremities with 10.2%. Furthermore, the tumor was identified in ocular, gastric, rectal, hepatic, ovary, ileal, and urethral localizations. The most common histopathological type was nodular malignant melanoma with 35.8% (n = 28) in patients with accessible data. Additionally, there was superficial MM in six patients, pigmented and acral lentiginous MM in two patients each, and amelanotic MM in one patient. Based on TNM classification, 26.9% (n = 21) of the patients were stage 4, 17.9% (n = 14) were stage 3, and 14.1% (n = 11) were stage 2. Given the patient distribution based on the Clark and Breslow staging, 26.9% (n = 21) of the patients were Clark stage 4, which was the most common, and 37.1% (n = 29) were Breslow stage 4. There was metastasis in 61.5% (n = 48) of the patients. The most common region for metastasis was the lungs with 28.2% (n = 22).

**Figure 3:**
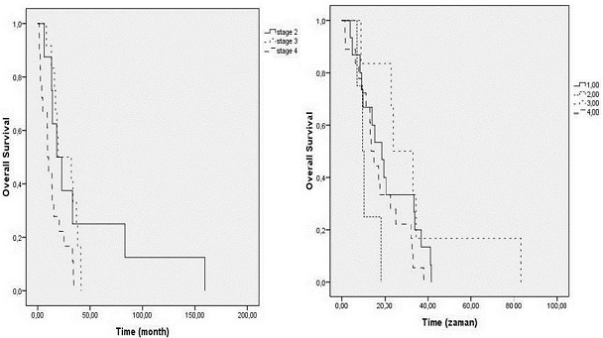
Survival rates according to tumor location and TNM staging

Furthermore, nine patients each (11.5%) had metastasis in the liver, bone, and lymph node, seven patients had metastasis in the brain, two patients had metastasis in the adrenals, and one patient each had metastasis of the ovaries, spinal, tonsillar, and appendix metastasis. Again, there was metastasis in multiple foci in 11 patients. Demographics and pathological data of patients are summarized in Table 1. Mean survival time after diagnosis was 14.9 months. The median overall survival time was 16.9 months in females and 12.6 months in males. Stage was found as a prognostic factor for survival in melanoma patients. Given these data, it seems that patients are generally diagnosed late and therefore treated late. Consequently, this results in increased MM mortality and morbidity. It was established that 52 (66.6%) of the patients included in the study died. Median overall survival in all patients was 14.9 months (95% CI 10.9 - 18.8 months). Median overall survival time was 16.9 months (95% CI 9.8 - 24.1) in females and 12.6 months (95% CI 7.9 - 17.4) in males (p = 0.055). Median survival times by stage were 18.1 months at stage 2 (95% CI 5.6 - 30.5), 19.4 months at stage 3 (95% CI 0.0 - 42.5), and 9.3 months at stage 4 (95% CI 6.9 - 11.7). As is seen, there was a significant reduction in survival time at stage 4 (p = 0.012, Figure 3). By age, median time was 12.6 months (95% CI 6.4 - 18.8) under the age of 50 years and 15.2 months (95% CI 10.3 - 20.2) above the age of 50 years (p = 0.195). By tumor localization, median time was 18.4 months (95% CI 11.6 - 25.2) for head-neck localization, 9.6 months (95% CI 6.3 - 13.0) for the trunk, 23.9 months (95% CI 11.7 - 36.1) for the upper extremities and 13.6 months (95% CI 10.1 - 17.1) for the lower extremities. Based on these results, there was a reduction in the limit of statistical significance level for trunk localization (p = 0.056) (Figure 3). In the multivariate Cox analysis, only the stage from these parameters statistically significantly affected survival [Odds ratio (OR): 0.54; (95% CI 0.16-1.82, p = 0.02, Table 2. There was no statistically significant difference between tumor localization, histopathological type, Clark and Breslow staging, metastasis regions, and length of life by gender.

**Table 1:** Demographic and pathological characteristics of patients

	Patient (n)	%
**Site**		
Skin	61	78.2
Organ	7	8.9
Mucosal	2	2.5
Ocular	2	2.5
**Gender**		
Male	44	56.4
Female	34	43.6
**Anatomic Location**		
Lower extremities	23	29.4
Head-Neck	20	25.6
Trunk	10	12.8
Other	25	32
**Histopathological type**		
Nodular MM	28	35.8
Superficial MM	6	7.6
Other	5	6.4
Unknown MM	39	50
**Stage**		
**Clark**		
2	4	5.1
3	7	8.9
4	21	26.9
5	3	3.8
Unknown	43	55.1
**Breslow**		
2	1	1.2
3	4	5.1
4	29	37.1
Unknown	44	56.4
**TNM**		
2	11	14.1
3	14	17.9
4	21	26.9
Unknown	32	41
**Metastasis site**		
Lung	22	28.2
Liver	9	11.5
Lymph node	9	11.5
**Histopathologic findings**	**Positive patients**	**Total**
Perineural invasion	1	18
Lymphovascular invasion	6	19
Satellite and intransit metastasis	2	14
Ulceration	10	21

**Table 2:** Factors of determining survival (according to the Cox regression model)

	p	OR	%95 CI	
			Lower bound	Upper bound
**Age**	0.989	1.00	0.964	1.04
**Gender**	0.427	1.44	0.584	3.56
**Stage**	0.033	2.31	1.7	5.00
**Tumor location**	0.678	1.18	0.54	2.58

## Discussion

A malignant melanoma is a tumor with poor prognosis, which develops mostly from the skin, but also from other tissues containing melanocyte such as mucosa, eye, and meninges. The study by Chang et al. reported that 91.2% of the patients had skin-based melanoma, 5.3% had ocular melanoma, 1.3% had mucosal melanoma, and 2.2% had melanoma of unknown primary origin [8]. In the present study, the most common was cutaneous MM (78.2%). Most of MMs are seen in adults; however, there may be cases in children, especially developing from congenital nevi. Three hundred fifty-five melanoma patients are under the age of 45 years. The mean age at diagnosis is 52 years. The female/male ratio is approximately 1:1 in USA and Australia [1, 2, 9]. Studies from Turkey reported the mean age to be 45-60 years. With a slightly higher male number in male/female ratio, Simsek et al. found a higher number of females in their study [10–13]. Studies from Italy, Holland, Spain, and Portugal reported that patients were mostly within the age range of 40-70 years and MM was more common in females [14–17]. In Asia, Chi et al. reported that 82.2% of 522 MM patients were aged 65 years and above [3]. Seventy-five percent of our patients were aged 50-80 years and the male/female ratio was 1:1.29. Although the number of males was slightly higher, it was not statistically significant. Four histological subtypes of MM were defined as superficial spreading MM, nodular MM, lentigo MM, and acral lentiginous MM. Superficial malignant melanomas account for 70% of all melanomas. They are mostly located in the trunk in males and the legs in females. Nodular MM is the second most common type with 15-30%, mostly at the age of 50-60 years. It is the type with the poorest prognosis. It is mostly located in the trunk, head, and neck. It is generally diagnosed in the advanced stage. Lentigo MM accounts for 5-15% of melanomas and it is usually seen at the age of 60-80 years. Acral lentiginous MM is a very rare type (2-3%) and seen at the age of 60-70 years [1].

In Australia, MM was reported mostly as the superficial type, located in the trunk in males and the lower extremities in females [4, 5]. Studies from Europe (Italy, Holland, Spain, and Portugal) reported that the most common MM type was superficial with the most common tumor localization in the trunk and lower extremities [14–17]. Studies from Asia found acral lentiginous MM as the most common type and palmoplantar region as the most common localization [3]. The study by Moreno et al. from Brazil and studies from Argentina, Uruguay, and Chile found that the most common histological type was superficial MM and the most common localization was extremities and trunk in females and males [5, 18]. Studies from Turkey reported nodular MM as the most common MM type, whereas Tas et al. reported superficial MM as the most common type [10]. The most common localizations were the lower extremities and the head-neck region [10–13]. The present study found nodular MM as the most common type, consistent with studies from Turkey but different from studies from Europe and the US. In terms of localization, the most common region was the lower extremities and the head-neck region, consistent with the results of all studies. Concerning the localisation of the tumour, it was observed to involve different regions of the gastrointestinal system (GIS) (i.e. stomach, rectum, liver and ileum) following the involvement of extremities, head & neck and truncal region. Anorectal region is only the third to skin and eye as the most frequent locations of melanomas [19]. Primary malignant melanoma of the GIS is a rare occurrence. Lesions observed in the GIS usually represent the metastases of melanomas on the skin with no identifiable primary focus [20]. Melanoma of the stomach is one of the common types of melanomas that involve the GIS. The primary origin of a melanoma of the stomach is very unlikely and can be accepted only in the confirmed absence of any other primary lesions [21]. One of our patients had stomach involvement.

One of the histological characteristics of MM is Breslow thickness (invasion depth) and it is the strongest determinant for lifetime. Additionally, multiple histological characteristics should be specified, including Clark′s level of invasion, ulceration, presence of tumor-infiltrating lymphocytes, mm2/mitoses, regression, vascular invasion, perineural invasion, and microscopic satellites. For staging, the TNM (tumor-lymph node-metastasis) staging system is used, which was established by the AJCC [22]. In studies from the US and Europe, MM is usually diagnosed in the early stage. A study from the US reported that 62.6% of the patients were diagnosed at TNM stage 0 and 1. In studies from Europe, the most common were Breslow 1, Clark IV, and TNM stage 1 [14–17]. In Africa, most of the MM cases were found at TNM stage 3 or higher [5]. In Brazil, Moreno et al. reported Breslow 1, Clark IV, and TNM stage 1 as the most common, whereas Clark 3-4 was the most common in Australia [18]. In studies from Asia, the most common were Breslow 1, Clark stage 3, and TNM stage 2 [3]. Studies from Turkey reported that most of the patients were at Breslow stage 3-5, Clark stage IV, and TNM stage 3-4 [10–13]. In the present study, the findings for Breslow 4, Clark IV, and TNM stage 4 were consistent with data reported from Turkey. These results suggest that MM is diagnosed late in Turkey compared to other countries and developed countries in particular. First metastasis in MM is frequently sentinel by lymphatic spread, i.e. to the first lymph node along the tumor′s lymphatic drainage. In the advanced stage of the disease, the skin, soft tissue, lung, liver, brain, bone, and bowel metastases are common sites through hematogenous spread. It rarely metastasizes to heart and breast [9, 22]. As reported by Tas et al. lung metastasis was the most common site in our patients [10].

## Conclusion

Malignant melanoma data are also important for the optimal utilization of effective methods and healthcare resources to prevent the disease. In order to minimize MM mortality and morbidity, not only society but also the physicians from primary and secondary care hospitals should become familiar with melanoma, awareness should be raised, and the importance of UV exposure should be expressed. In this respect, a significant role falls into dermatologists and dermatology society in particular. The primary preventive strategy should aim for sun protection and avoiding sunburns, especially in children and adolescents. Additionally, individuals who are light-skinned and in the at-risk group should be periodically checked, these data should be recorded, and the patients and their families should be warned in this regard.

### What is known about this topic

- The melanoma incidence has shown a significant increase for the last few decades around the world;
- Seventy-five percent of deaths from skin cancers are due to melanoma. Due to the poor prognosis and fast course, it is also an important public health issue.

### What this study adds

- In order to minimize MM mortality and morbidity, not only the society but also physicians from primary and secondary care hospitals should become familiar with melanoma;
- Individuals who are light-skinned and in the at-risk group should be periodically checked, these data should be recorded, and the patients and their families should be warned in this regard.
